# Impaired Cerebral Autoregulation After Subarachnoid Hemorrhage: A Quantitative Assessment Using a Mouse Model

**DOI:** 10.3389/fphys.2021.688468

**Published:** 2021-06-08

**Authors:** Masayo Koide, Hannah R. Ferris, Mark T. Nelson, George C. Wellman

**Affiliations:** ^1^Department of Pharmacology, Larner College of Medicine, University of Vermont, Burlington, VT, United States; ^2^Vermont Center for Cardiovascular and Brain Health, Larner College of Medicine, University of Vermont, Burlington, VT, United States; ^3^Division of Cardiovascular Sciences, University of Manchester, Manchester, United Kingdom

**Keywords:** cerebral autoregulation, mice, endovascular perforation, microsphere, cerebral blood flow, quantification, laser Doppler flowmetry, subarachnoid hemorrhage

## Abstract

Subarachnoid hemorrhage (SAH) is a common form of hemorrhagic stroke associated with high rates of mortality and severe disability. SAH patients often develop severe neurological deficits days after ictus, events attributed to a phenomenon referred to as delayed cerebral ischemia (DCI). Recent studies indicate that SAH-induced DCI results from a multitude of cerebral circulatory disturbances including cerebral autoregulation malfunction. Cerebral autoregulation incorporates the influence of blood pressure (BP) on arterial diameter in the homeostatic regulation of cerebral blood flow (CBF), which is necessary for maintaining constant brain perfusion during physiological swings in systemic BP. In this study, we quantitatively examined the impact of SAH on cerebral autoregulation using a mouse endovascular perforation model and a newly developed approach combining absolute and relative CBF measurements. This method enables a direct quantitative comparison of cerebral autoregulation between individual animals (e.g., SAH vs. control or sham-operated mice), which cannot be done solely using relative CBF changes by laser Doppler flowmetry. Here, absolute CBF was measured *via* injection of fluorescent microspheres at a baseline BP. In separate groups of animals, *in vivo* laser Doppler flowmetry was used to measure relative CBF changes over a range of BP using phlebotomy and the pressor phenylephrine to lower and raise BP, respectively. Absolute CBF measurements from microspheres were then used to calibrate laser Doppler measurements to calculate the relationship between CBF and BP, i.e., “cerebral autoregulation curves.” Un-operated and sham-operated groups exhibited similar cerebral autoregulatory curves, showing comparable levels of relatively constant CBF over a range of BP from ~80 mmHg to ~130 mmHg. In contrast, SAH animals exhibited a narrower autoregulatory range of BP, which was primarily due to a decrease in the upper limit of BP whereby cerebral autoregulation was maintained. Importantly, SAH animals also exhibited a marked decrease in CBF throughout the entire range of BP. In sum, this study provides evidence of the dramatic reduction in cortical CBF and the diminished range of autoregulation after SAH. Furthermore, this novel methodology should pave the way for future studies examining pathological mechanisms and/or therapeutic strategies targeting impaired cerebral autoregulation, a pathology common to many cardiovascular and cerebrovascular disorders.

## Introduction

Subarachnoid hemorrhage (SAH), a devastating form of hemorrhagic stroke caused by cerebral aneurysm rupture (van Gijn et al., 2007; Athar and Levine, 2012), occurs in ≈30,000 people in the United States each year often resulting in death (6-month mortality rate: ≈50%) or severe disability (Sacco et al., 1984; Hop et al., 1997; Weir, 2002). Unfortunately, current treatments for SAH are limited and do little to improve outcomes (Bederson et al., 2009; Athar and Levine, 2012; Smetana et al., 2020). A common complication faced by these patients is delayed cerebral ischemia (DCI), a characteristic pathology unique to SAH patients leading to the development of serious neurological deficits days after the initial aneurysm bleed (Rouanet and Silva, 2019; Neifert et al., 2020). Traditionally, SAH-induced DCI has been attributed to cerebral vasospasm—severe and sustained constriction of large-diameter arteries on the brain surface (Ecker and Riemenschneider, 1951; Suzuki et al., 2021). Of great significance, a wealth of recent experimental and clinical studies has demonstrated a disconnection between large artery cerebral vasospasm and DCI. It is now appreciated that in the aftermath of SAH, multiple mechanisms contribute to DCI including inflammatory reactions, cortical spreading depolarization, secondary responses to acute pathological events known as early brain injury, microcirculation and blood-brain barrier disturbances, and cerebral autoregulation failure (Macdonald et al., 2007; Budohoski et al., 2013; Koide et al., 2013; Neifert et al., 2020; Suzuki et al., 2021).

Cerebral autoregulation is fundamental to the control of cerebral blood flow (CBF), maintaining relatively constant brain perfusion in the face of continual physiological fluctuations in systemic blood pressure (Paulson et al., 1990; Armstead, 2016; Silverman and Petersen, 2020). This phenomenon is principally achieved by pressure-dependent alterations in the resistance of small-diameter cerebral arteries and arterioles (Brayden et al., 2008; Silverman and Petersen, 2020), which is then fine-tuned by additional homeostatic mechanisms including neurogenic, hormonal, and metabolic responses (Koller and Toth, 2012). As first reported by Bayliss (1902), cerebral arteries possess a “myogenic response” or an intrinsic ability to raise vascular resistance *via* enhanced constriction in response to increases in systemic blood pressure and conversely dilate promoting enhanced blood flow when blood pressure drops. Since the initial report by Bayliss, the molecular mechanisms underlying myogenic tone have been extensively studied. Although the identity of pressure-sensing molecules within the vascular wall is still debated (Brayden et al., 2008, 2013; Mederos y Schnitzler et al., 2008; Gannon et al., 2015), the linkage between elevated intraluminal pressure, cerebral artery myocyte membrane potential depolarization, activation of voltage-dependent Ca^2+^ channels, and vasoconstriction has been clearly established (Knot and Nelson, 1998; Gollasch et al., 2000; Brayden et al., 2008).

Pressure-induced cerebral vasoconstriction is altered in a variety of pathological conditions. For example, cerebral artery myogenic tone is attenuated in animals with aging (Toth et al., 2013), ischemic stroke (Cipolla and Bullinger, 2008; Coucha et al., 2013), and traumatic brain injury (Villalba et al., 2014). Conversely, pressure-induced constriction is enhanced in hypertensive animals (Toth et al., 2013; Pires et al., 2015) and after hemorrhagic stroke such as SAH (Ishiguro et al., 2002; Koide et al., 2011; Nystoriak et al., 2011). Moreover, SAH-induced augmentation of myogenic constriction has been observed in both brain surface arteries (Ishiguro et al., 2002, 2005, 2006) and parenchymal arterioles (Nystoriak et al., 2011; Wellman and Koide, 2013; Koide and Wellman, 2015). Considering the innate properties of cerebral vessels in response to intraluminal pressure, pathological alterations in the myogenic response are likely to play a large part in compromised cerebral autoregulation and decreased brain perfusion.

This study aimed to elucidate the impact of SAH on cerebral autoregulation using an endovascular perforation SAH model in mice. Previous studies (Huang et al., 1996; Niwa et al., 2002) have evaluated cerebral autoregulation experimentally using laser Doppler flowmetry, which provides measurements of relative changes in blood flow in arbitrary units. However, a limitation of studies relying solely on laser Doppler flowmetry is that comparisons of blood pressure-CBF relationships or “cerebral autoregulation curves” cannot be made between groups of animals having different basal CBF (e.g., between SAH and control animals). To circumvent this issue, we developed an approach that combined microsphere methodology to obtain absolute values of baseline CBF and laser Doppler flowmetry measurements of relative CBF over a wide range of systemic blood pressures. Compared to control and sham-operated animals, we found SAH mice to have a narrowed autoregulatory range with marked reductions in CBF over the range of blood pressures we examined (40–190 mmHg). Our findings shed light on SAH-induced decreases in CBF that likely contribute to DCI and the development of neurological deficits. Importantly, the experimental methods and analytical approaches described in this work can also be applied to the study of cerebral autoregulatory deficits in the broad scope of animal models of disease.

## Materials and Methods

### Mouse SAH Model

SAH was induced in 3–5-month-old male C57BL/6 mice (The Jackson Laboratory) using an endovascular perforation model as previously described (Balbi et al., 2017a,b). Briefly, under isoflurane anesthesia (5.0% induction and 2.0% maintenance), a 5–0 monofilament was introduced via the left external carotid artery into the internal carotid artery and advanced to the anterior cerebral artery (ACA)-middle cerebral artery (MCA) bifurcation, where resistance was encountered. Further advancement of the suture (~3 mm) from the ACA-MCA bifurcation caused vascular perforation and SAH. The suture was then immediately withdrawn, and the external carotid artery ligated. Animals in the sham surgery group underwent the entire procedure except for suture perforation (i.e., no further advancement of the suture from ACA-MCA bifurcation). As SAH was induced on the left hemisphere of the brain, with un-operated animals, the left and right sides of the cortex were used as “ipsilateral” and “contralateral” cortex, respectively. Cortical CBF was monitored in sham-operated and SAH animals by laser Doppler flowmetry with a flow probe (PeriMed, probe 418) glued directly to the exposed, intact skull as shown in Supplementary Figure 1. Intracranial pressure was not monitored during SAH induction, thus maintaining an intact dura mater. After regaining consciousness, animals were returned to their original cage within the animal care facility. Buprenorphine (0.05 mg/kg) was given before the surgery and 12 h after the surgery as an analgesic. Cerebral autoregulation was evaluated 24 h after the surgery, a time frame corresponding to the peak in vascular pathology observed in rodent SAH models (Pappas et al., 2015; Balbi et al., 2017b). After every experiment, the animal’s brain was removed and a blood clot confirmed on the brain surface to verify the successful induction of SAH. All procedures were conducted in accordance with the Guide for the Care and Use of Laboratory Animals (eighth edition, 2011), ARRIVE (Animals in Research: Reporting In Vivo Experiments) guidelines, and followed protocols approved by the Institutional Animal Care and Use Committee at the University of Vermont.

### Laser Doppler Flowmetry

The relationship between relative changes in CBF and systemic blood pressure was evaluated under the combined anesthesia of urethane (750 mg/kg, i.p.) and α-chloralose (50 mg/kg, i.p.). The use of urethane and α-chloralose as anesthetic agents has previously been shown to have a minimum effect on CBF and cerebral autoregulation in mice (Niwa et al., 2002). As intracranial pressure significantly contributes to determine cerebral perfusion pressure and subsequently affects CBF, it is important to evaluate cerebral autoregulation under the condition of intact intracranial pressure (Balbi et al., 2017b). Thus, relative changes of CBF within the ipsilateral cortex were measured by laser Doppler flowmetry using a single fiberglass microtip probe (PeriMed, probe 418) that was directly attached to the intact skull, enabling intracranial pressure to remain unaltered. Systemic blood pressure was simultaneously measured through a catheter inserted into a femoral artery and was controlled by intraperitoneal administration of the α-1 adrenergic receptor agonist phenylephrine or phlebotomy using an intravenous catheter to increase and decrease blood pressure, respectively. Body temperature was maintained at 37°C using a heating pad thermostatically controlled by a rectal probe.

### Quantification of CBF Using Microspheres

Tissue perfusion in the ipsilateral and contralateral cerebral cortex, cerebellum, spleen, and kidney was quantified in separate groups of animals using fluorescently tagged microspheres (De Visscher et al., 2006; Reading and Brayden, 2007; Wang et al., 2010). In brief, control, sham-operated, and SAH animals were anesthetized with urethane (750 mg/kg, i.p.) and α-chloralose (50 mg/kg, i.p.), and blood pressure was measured through a cannula inserted into a femoral artery. Fluorescently tagged microspheres (~15 μm in diameter and ~1 × 10^6^ microspheres suspended 0.1 ml saline, Invitrogen) were injected through the left ventricle of the heart, and a reference blood sample (~0.1 ml) was collected from a femoral artery cannula 1 min after microsphere injection. Animals were then immediately decapitated, and tissues collected, weighed, and stored at −80°C until assayed. Following previously established procedures (De Visscher et al., 2006; Reading and Brayden, 2007; Wang et al., 2010), tissues were alkaline lysed and the fluorescent dye was extracted from microspheres with 2-ethoxyethyl acetate. The fluorescent intensity of solutions was measured by a Synergy^™^ H4 Hybrid Multi-Mode Microplate Reader (BioTek). Tissue blood flow was calculated using the following equation: Blood flow (ml/100 g tissue/min) = [(Fluorescent intensity in tissue/Fluorescent intensity in reference blood) × blood sampling rate (ml/min)] × 100/tissue weight (g) as shown by De Visscher et al. (2006); Reading and Brayden (2007) and Wang et al. (2010).

### Analysis of Cerebral Autoregulation

**Figure 1:**
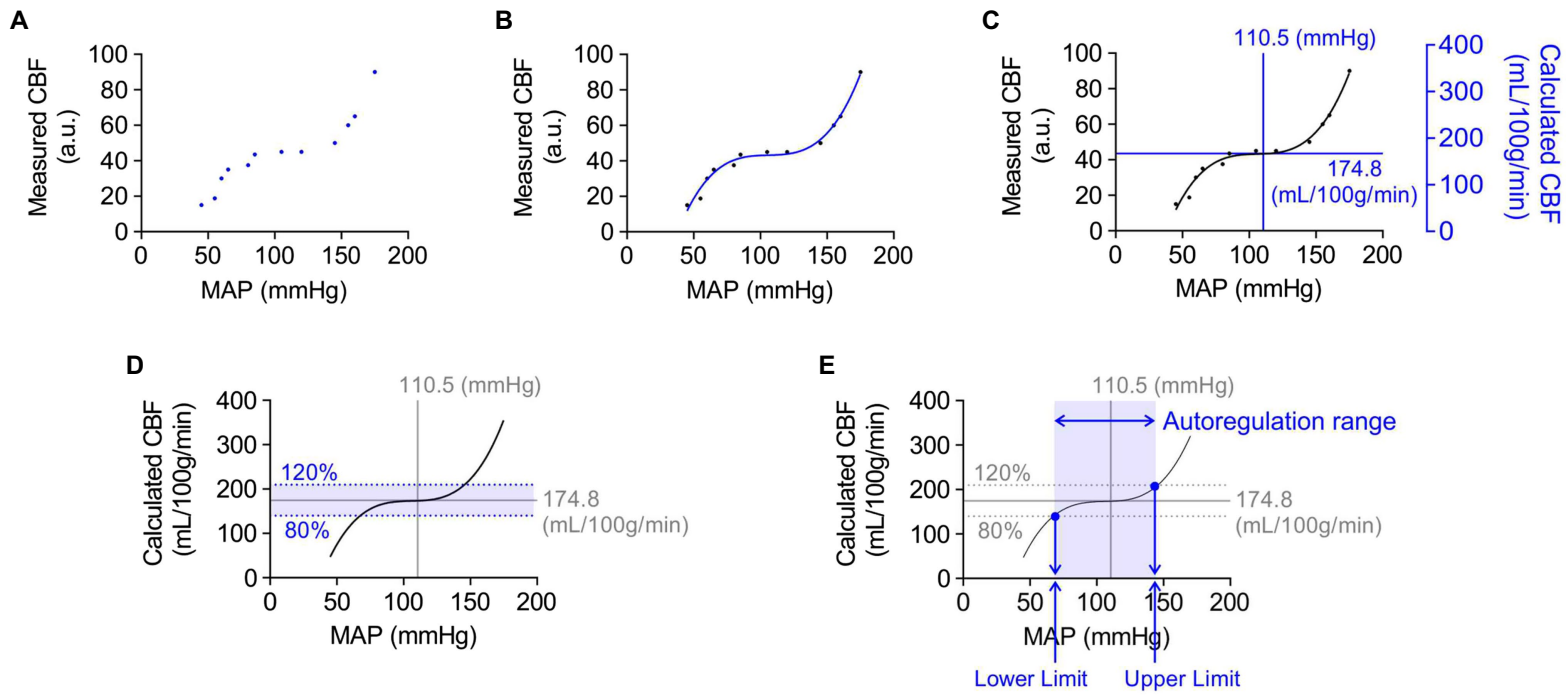
Example of step-by-step analysis of cerebral autoregulation. Blue dots, lines, and text represent new steps included in successive panels. **(A)** Scatter plot of mean arterial pressure (MAP, mmHg) and cerebral blood flow (CBF) measured by laser Doppler flowmetry (a.u., arbitrary unit). The combinations of each MAP and CBF data points were simultaneously obtained from the same animal. **(B)** Regression curve showing the relationship between MAP and CBF. A non-linear regression curve was fitted by third-order polynomial function. **(C)** Overlaying the scatter plot with the values of MAP and quantified CBF obtained from animals in the same group. Absolute CBF value on the Y-axis was used to quantify relative changes in blood flow obtained using laser Doppler flowmetry. **(D)** Definition of the cerebral autoregulatory range. The range within a 20% increase or decrease of CBF was considered as the limits of cerebral autoregulation. **(E)** Systemic blood pressure values at the upper and lower limits of cerebral autoregulation. The blood pressure limits of autoregulation were determined using values, shown as blue dots, which crossed the regression curve at the upper or lower limit of cerebral autoregulation. The difference in blood pressure between the upper and lower limits of CBF autoregulation was considered as the autoregulation range (mmHg).

Figure 1 describes the step-by-step approach used in this study for the analysis of cerebral autoregulation curves. First, scatter plots of simultaneous measures of relative CBF (arbitrary units, a.u.; obtained using laser Doppler flowmetry) versus mean arterial blood pressure (mmHg) were generated for individual animals (Figure 1A). These data points were then fitted with a non-linear regression curve by a third-order polynomial function (Figure 1B). As laser Doppler flowmetry provides relative changes in blood flow (a.u.), these measurements were then converted into values of absolute CBF (ml/100 g tissue/min) by calibration with empirically determined measures of tissue perfusion at baseline systemic blood pressure obtained from fluorescent microspheres studies using separate groups of similarly treated animals (Figure 1C). We defined cerebral autoregulation as CBF within ±20% of basal CBF based on the previous studies (Banaji et al., 2005; van Beek et al., 2008). Thus, the upper and lower limits of CBF autoregulation would be 80 and 120% of baseline CBF (quantified using microspheres), respectively (Figure 1D). These upper and lower autoregulatory limits were calculated using a non-linear regression curve fitted by the third-order polynomial function of MAP-CBF plots. Systemic blood pressure at the upper and lower limits of CBF autoregulation was determined from the intersection of fitted cerebral autoregulatory curves with lines depicting 120 and 80% of baseline CBF (Figure 1E). The difference in blood pressure between the upper and lower limits of CBF autoregulation was considered the autoregulation blood pressure range (mmHg).

### Reagents

Fluorescence-tagged microspheres were purchased from Invitrogen (cat# F8844). All other chemicals and reagents were obtained from Sigma-Aldrich.

### Statistics

Data are presented as mean ± standard error of the mean. Statistical analysis was accomplished by GraphPad Prism 8 software using one-way ANOVA followed by Tukey’s multiple comparisons test (Tukey’s). *p* < 0.05 was considered statistically significant.

## Results

### Subarachnoid Hemorrhage Decreases Basal Cerebral Blood Flow

**Figure 2:**
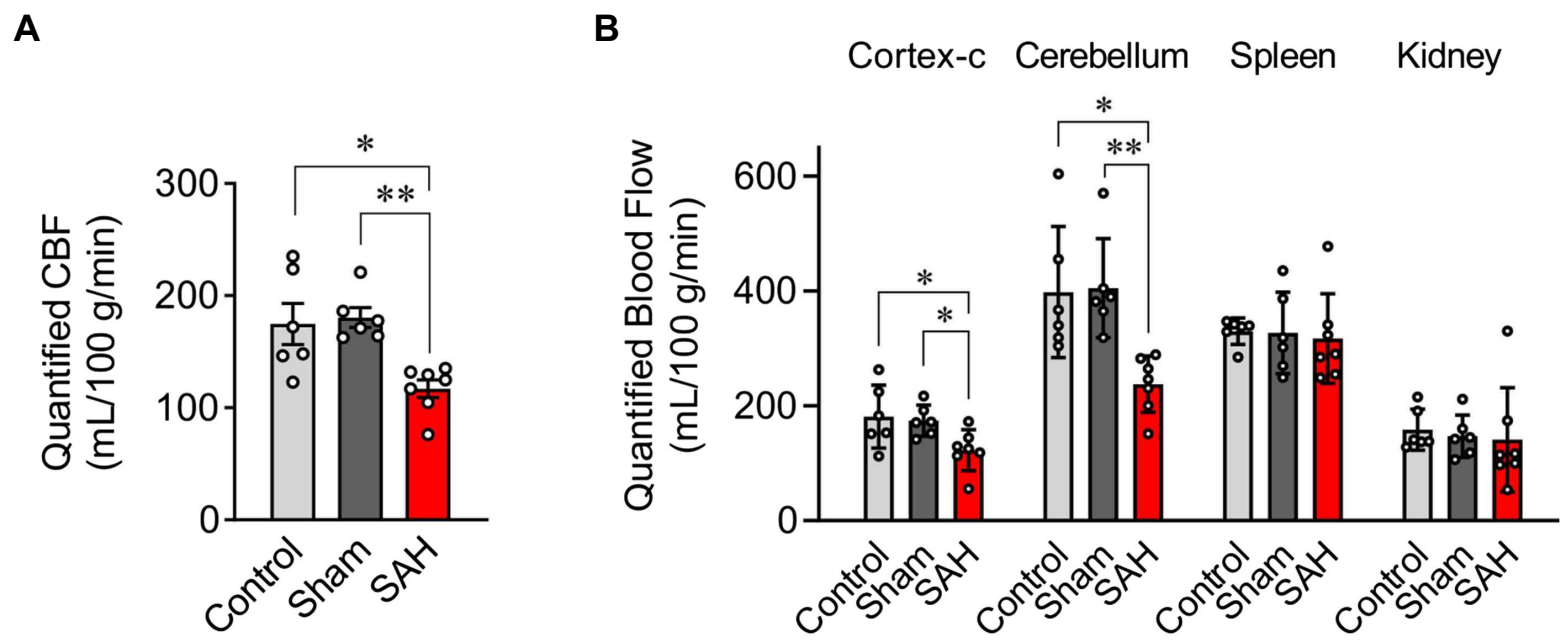
Quantification of blood flow using the fluorescent-microsphere method. **(A)** Quantified (CBF) in the ipsilateral cortex of the mouse brain from un-operated (shown as “Control”), sham-operated (shown as “Sham”), and SAH model animals (*n =* 6–7). **(B)** Tissue perfusion in the contralateral cortex of the brain (cortex-c), cerebellum, spleen, and kidney (*n =* 6–7). Sham and SAH groups were measured at 24 h after the surgery. ^*^p < 0.05, ^**^p < 0.01 between groups, by one-way ANOVA followed by Tukey’s multiple comparison test.

Utilizing fluorescently tagged microspheres (De Visscher et al., 2006; Reading and Brayden, 2007; Wang et al., 2010), tissue perfusion was quantified within the ipsilateral and contralateral cerebral cortex, cerebellum, spleen, and kidney in anesthetized un-operated control, sham-operated, and SAH animals. Mean arterial blood pressure and other physiological parameters such as body weight, temperature, heart rate, and blood pO_2_, pCO_2_, and pH were not different between treatment groups (Table 1). As blood pressure was not manipulated in these animals, levels of tissue perfusion determined in this experimental series are considered measures of basal tissue blood flow. Basal CBF in ipsilateral cortex was markedly decreased in SAH mice (116.9 ± 7.9 ml/100 g/min, *n =* 7) compared to control (174.8 ± 18.5 ml/100 g/min, *n =* 6) and sham-operated animals (180.5 ± 8.9 ml/100 g/min, *n =* 6, Figure 2A). Although the reduction in basal CBF was more pronounced on the ipsilateral or “hemorrhage” side of brain cortex, SAH also diminished basal CBF in the contralateral cortex (SAH: 119.8 ± 11.9 ml/100 g/min, *n =* 7 vs. control: 181.2 ± 22.4 ml/100 g/min, *n =* 6 vs. sham-operated animals: 177.5 ± 11.5 ml/100 g/min, *n =* 6, Figure 2B). Blood perfusion in the cerebellum was also significantly reduced in SAH animals (237.7 ± 18.5 ml/100 g/min, *n =* 7) vs. control (398.1 ± 46.6 ml/100 g/min, *n =* 6) and sham-operated animals (404.9 ± 35.2 ml/100 g/min, *n =* 6). Considering that there were no differences in the wet weight of brain tissue among groups (Table 1), the observed SAH-induced decreases in CBF cannot be attributed to increased water content (i.e., edema). Further, SAH did not impact blood flow to the spleen or kidneys (Figure 2B); however, spleen wet weight was significantly decreased in the SAH group. Collectively, these data demonstrate that SAH caused a reduction in basal CBF, which was most pronounced in the ipsilateral brain cortex.

**Table 1:** Physiological parameters in un-operated, sham-operated, and SAH model mice used for CBF quantification.

	Control (*n =* 6)	Sham (*n =* 6)	SAH (*n =* 7)
Body Weight (g)	28.7 ± 1.2	29.7 ± 1.1	25.7 ± 1.6
Blood Pressure (mmHg)	110.5 ± 6.8	103.7 ± 8.0	101.8 ± 7.4
Heart Rate (bpm)	518 ± 19	516 ± 22	524 ± 24
Body Temperature (°C)	37.0 ± 0.1	36.9 ± 0.1	36.9 ± 0.1
Blood Gas			
pO_2_ (mmHg)	91.7 ± 2.7	89.3 ± 2.7	90.6 ± 1.7
pCO_2_ (mmHg)	46.5 ± 1.5	47.0 ± 2.0	45.0 ± 1.7
pH	7.303 ± 0.016	7.323 ± 0.020	7.308 ± 0.018
Tissue Weight (mg)			
Ipsilateral Cortex	120.8 ± 4.5	115.2 ± 4.4	128.3 ± 6.4
Contralateral Cortex	118.3 ± 4.6	121.7 ± 4.9	127.0 ± 9.4
Cerebellum	56.0 ± 3.6	57.7 ± 1.5	59.7 ± 2.6
Spleen	69.3 ± 4.5	71.2 ± 4.6	53.1 ± 2.0^*^ ^‡‡^
Kidney	167.3 ± 4.3	203.7 ± 18.7	176.4 ± 8.8

* *p < 0.05 vs. un-operated control group*.

‡‡ *p < 0.01 vs. sham-operated group, by one-way ANOVA followed by Tukey’s multiple comparison test*.

### The Delineation of Cerebral Autoregulation Curves

**Figure 3:**
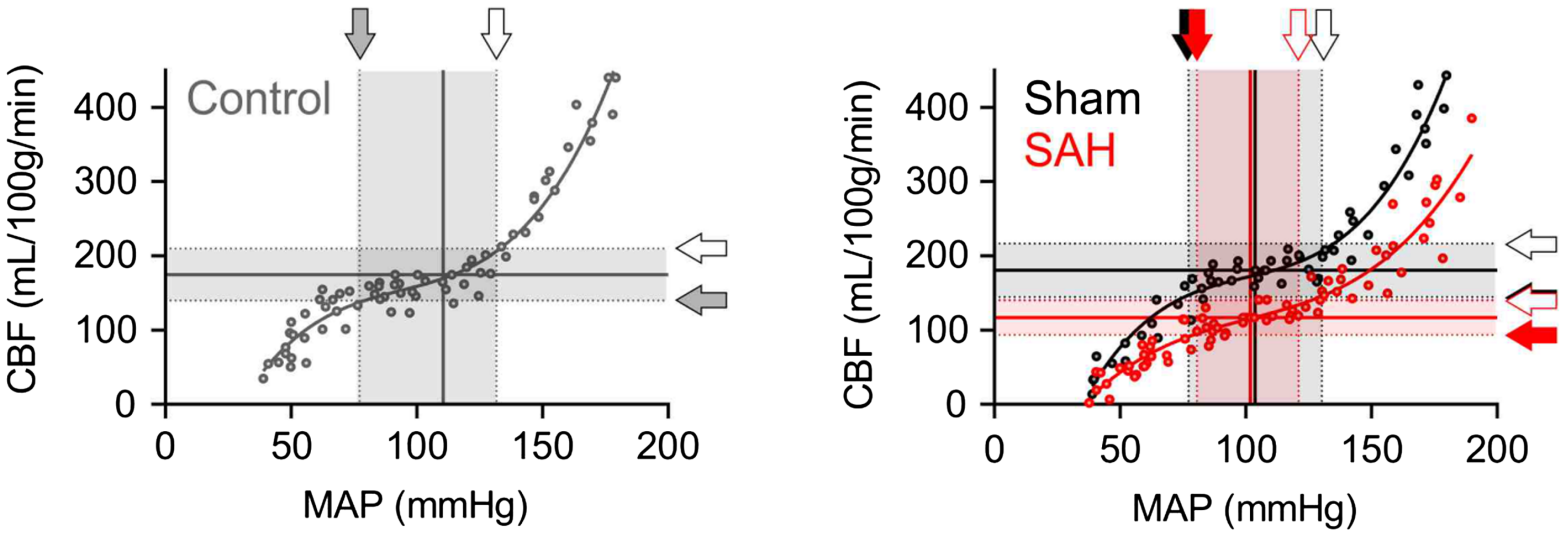
The downward shift of the cerebral autoregulation curve in SAH model animals. Cerebral autoregulation curves were obtained by the analysis procedure described in the “Material and Methods” section and in Figure 1, using all data points from five un-operated (shown as “Control” in gray), four sham-operated (shown as “Sham” in black), and six SAH model animals (shown in red). Sham and SAH groups were examined at 24 h after the surgery. Shaded arrows indicate the lower limits of cerebral autoregulation, and opened arrows point the upper limits of cerebral autoregulation.

**Figure 4:**
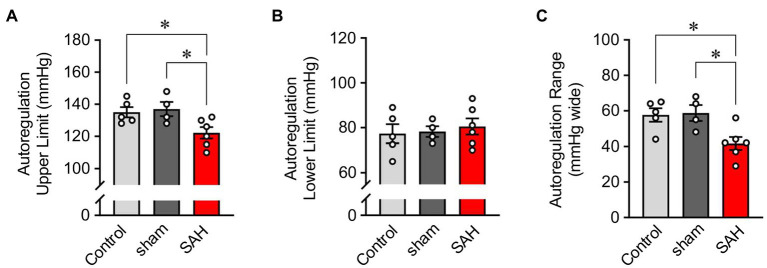
The narrowed range of cerebral autoregulation in SAH model animals. The upper- **(A)** and lower-limits blood pressures **(B)** and the range **(C)** of cerebral autoregulation in un-operated (shown as “Control”), sham-operated (shown as “sham”), and SAH model animals (*n =* 4–6). The upper and lower limits CBF of cerebral autoregulation were, respectively, 139.7 and 209.4 ml/100 g/min in un-operated animals, 144.2 and 216.2 ml/100 g/min in sham-operated animals, and 93.7 and 140.5 ml/100 g/min in SAH model animals (*n =* 4–6 per group). Sham and SAH groups were examined at 24 h after the surgery. ^*^p < 0.05 between groups, by one-way ANOVA followed by Tukey’s multiple comparison test.

Cerebral autoregulation (Paulson et al., 1990; Armstead, 2016; Silverman and Petersen, 2020) or the ability to maintain relatively constant CBF within the physiological range of systemic blood pressure is frequently examined using laser Doppler flowmetry (Huang et al., 1996; Niwa et al., 2002). However, as laser Doppler flowmetry provides only measures of relative changes in blood flow, comparisons of cerebral autoregulation between individuals or animals are difficult. Such comparisons are especially problematic when using groups that have varying levels of basal CBF, as is the case in this study, which compares control and SAH animals. Therefore, as described in the Methods section and shown in Figure 1, we established an approach that uses CBF quantified at a specific blood pressure with microspheres to convert relative blood flow changes obtained using laser Doppler flowmetry into estimates of actual blood flow. In essence, we have used microsphere measurements of absolute CBF to calibrate laser Doppler CBF measurements obtained at the same blood pressure, which enables the conversion of laser Doppler measurements obtained at other blood pressures into estimates of actual blood flow. As shown in Figure 3, values of mean arterial pressure (MAP, mmHg) and CBF (ml/100 g tissue/min) were plotted and fitted with non-linear regression to obtain the MAP-CBF relationship or “cerebral autoregulatory curve.” Consistent with the previous observations (Niwa et al., 2002), with un-operated control animals, the MAP-CBF relationship exhibited a “plateau phase” or autoregulatory range whereby relatively constant CBF was maintained between the range ~80 mmHg and ~130 mmHg. At blood pressures above or below these limits, the relationship between CBF and MAP was linear, with CBF declining with reductions in MAP below the autoregulatory range and increasing with elevations in MAP above the autoregulatory range. Analysis of the MAP-CBF regression curve from un-operated control animals provided blood pressure values for the upper and lower limits of cerebral autoregulation of 135.0 ± 3.2 mmHg and 77.3 ± 4.2 mmHg, respectively (*n =* 5, Figures 4A, B). In un-operated control animals, the width of the autoregulation range, or the difference between the upper and lower limits of cerebral autoregulation, was 57.7 ± 3.8 mmHg wide (*n =* 5, Figure 4C). The MAP-CBF relationship in sham-operated animals was not significantly different from that of un-operated control animals, having upper and lower limits cerebral autoregulation of 137.0 ± 4.4 mmHg and 78.2 ± 2.4 mmHg, respectively. The autoregulatory range was 58.8 ± 4.8 mmHg wide (*n =* 4) in the sham-operated group, which was not statistically different compared to the control group. These results demonstrate an approach enabling comparisons of MAP vs. CBF relationships between groups of animals and that the sham-operation procedures performed in this study do not impact cerebral autoregulation.

### SAH Causes a Downward Shift in CBF and a Narrowed Range of Cerebral Autoregulation

The relationship between blood pressure and cortical CBF was also examined in SAH animals 24 h after hemorrhage was induced *via* endovascular perforation at the Circle of Willis. CBF was dramatically decreased in SAH animals across the entire range of blood pressures examined, i.e., between 40 mmHg and 190 mmHg (Figure 3). Interestingly, SAH animals still exhibited cerebral autoregulation as the MAP-CBF relationship exhibited a plateau phase (i.e., relatively constant CBF) over a range of systemic blood pressure, although absolute CBF was decreased by ≈33% compared to control animals. The autoregulation range, or “the width” of plateau phase, was also significantly narrowed in SAH mice (41.7 ± 3.6 mmHg wide, *n =* 6) compared to un-operated control and sham-operated animals. Analysis of the MAP-CBF relationship curve indicates that SAH impacts the upper limit (122.2 ± 3.5 mmHg, *n =* 6, *p* < 0.05), rather than the lower limit (80.5 ± 3.6 mmHg, *n =* 6, *p* > 0.05), of cerebral autoregulation (Figure 4). These findings demonstrate that SAH causes a marked reduction in CBF across a broad range of systemic blood pressures despite the cerebral vasculature maintaining a degree of autoregulation.

## Discussion

The overall metabolic demand of the brain, though significant, varies little over the course of a 24-h day. Thus, a relatively constant flow of nutrient-supplying blood is required to maintain the central nervous system healthy. Without cerebral autoregulation, or the intrinsic ability of cerebral arteries and arterioles to tune vascular resistance in response to fluctuations in blood pressure, the constancy of CBF would be lost and CBF would instead rise or fall proportionately with changes in cardiac output. Volatilities in CBF would translate into periods of hypoperfusion promoting cerebral ischemia and/or hyperperfusion potentially leading to increased intracranial pressure, cerebral edema, and structural damage to the vasculature and blood-brain barrier. Defects in cerebral autoregulation have been linked to a variety of pathologies including SAH; however, quantitative assessments are scarce owing to the widespread use of laser Doppler flowmetry, which provides only measures of relative changes in CBF within an individual or animal. Here, we report the development of an approach that combines laser Doppler flowmetry with microsphere measurements of absolute CBF, enabling direct comparisons of cerebral autoregulatory function between SAH, sham-operated, and un-operated mice. Our findings demonstrate that although SAH animals exhibit a characteristic cerebral autoregulatory curve, two marked quantitative differences are apparent in the relationship between systemic blood pressure and CBF when compared to sham-operated and un-operated groups. First, the autoregulation range, or “the width” of the plateau phase, was significantly narrowed in SAH mice reflecting a reduction in the upper limit of blood pressures able to maintain constant CBF. Secondly, and perhaps more importantly, despite exhibiting an ability to autoregulate blood flow, CBF was decreased by greater than 30% in SAH animals at blood pressures ranging from 60 mmHg to 180 mmHg. These findings provide valuable insights into cerebrovascular defects that likely contribute to SAH-induced (DCI) and underscore the value of quantitative approaches that enable evaluation of the relationship between systemic blood pressure and CBF.

For CBF quantification, we adapted fluorescent microsphere methodology widely used in rats (De Visscher et al., 2006; Reading and Brayden, 2007; Wang et al., 2010). Consistent with the previous studies in SAH model rats (Wang et al., 2010; Gong et al., 2019) and clinical observations in humans (Heilbrun et al., 1972; Granowska et al., 1980), we detected a significant decrease in basal CBF in SAH mice. Using these quantified values of basal CBF, relative changes in blood flow, obtained using laser Doppler flowmetry, were converted into absolute values of blood flow (i.e., ml/100 g tissue/min) and plotted versus the MAP at which the measurement was obtained. The CBF-MAP correlation plots exhibited a triphasic shape with a plateau phase in all three groups, characteristic of cerebral autoregulation (Figure 3). Although cerebral autoregulation was apparent in SAH animals, the ability to compare estimates of absolute CBF across groups revealed a striking observation—CBF was markedly reduced over a broad range of blood pressures in SAH animals, indicative of cerebral hypoperfusion. Cerebral ischemia is a major contributor to the high rates of morbidity and mortality occurring in SAH patients in the days following aneurysm rupture. For several decades, it was thought that SAH-induced DCI resulted solely from narrowing or “vasospasm” of large-diameter brain surface arteries (Pluta et al., 2009; Crowley et al., 2011), which play a minimal role in cerebral autoregulation (Budohoski et al., 2013). However, it is now appreciated that enhanced constriction of small-diameter arteries and arterioles, including those within the brain parenchyma contribute to cerebral ischemia in the aftermath of SAH. Potential agents promoting enhanced vasoconstriction after SAH include blood cell breakdown products, such as oxyhemoglobin, which is released during the lysis of clotted red blood cells into cerebrospinal fluid in the days after SAH (Macdonald and Weir, 1991; Macdonald et al., 2007; Pluta et al., 2009). Oxyhemoglobin is not only a robust vasoconstrictor of cerebral arteries and arterioles (Ishiguro et al., 2006, 2008) but also enhances myogenic tone (Ishiguro et al., 2002), the primary mechanism underpinning cerebral autoregulation (Brayden et al., 2008; Silverman and Petersen, 2020). Although further research is required, vasoactive agents that enhance myogenic tone including oxyhemoglobin, and possibly other blood cell breakdown products, likely contribute to decreased CBF after SAH.

Interestingly, we still observed cerebral autoregulation in SAH animals, despite decreased CBF over the physiological range of blood pressures. Our previous studies using both a rabbit double-injection SAH model (Ishiguro et al., 2002) and a rat double-injection SAH model (Nystoriak et al., 2011) demonstrated that cerebral arteries/arterioles developed intrinsic pressure-induced constriction (myogenic tone), as do arteries/arterioles from healthy animals. However, pressure-induced constriction was significantly greater in cerebral arteries/arterioles from SAH model animals compared to sham-operated animals. Considering that this intrinsic vasoconstriction is a key mechanism underlying cerebral autoregulation, the above-mentioned data support our current findings that: (1) SAH animals show cerebral autoregulation and (2) CBF is decreased over the physiological range of blood pressure. We also report a narrowing in the autoregulatory range of blood pressures in SAH animals. Using non-linear regression curve fitting of MAP-CBF plots, upper and lower limits of cerebral autoregulation were defined as blood pressures where CBF exceeded a 20% increase or decrease from basal values, respectively. The autoregulatory range was calculated as the difference between these boundaries. In healthy adult humans, the autoregulatory range has been estimated to be ≈100 mmHg with a lower MAP limit of ≈60 mmHg and an upper MAP limit of ≈160 mmHg (Armstead, 2016). Here, in un-operated control animals, the autoregulatory range was 58 mmHg wide, which is comparable to the previous studies (~60 mmHg) in awake (Lacombe et al., 2005) or urethane-chloralose anesthetized (Niwa et al., 2002) mice. Our results indicate that SAH narrows the autoregulatory range chiefly by reducing the upper limit of cerebral autoregulation. Precise molecular mechanisms controlling the lower and upper limits of cerebral autoregulation are unclear. However, *ex vivo* studies using isolated cerebral arteries demonstrate a phenomenon referred to as “forced dilatation,” where active vasoconstriction is progressively lost at intravascular pressures exceeding ≈140 mmHg (Ishiguro et al., 2002; Osol et al., 2002). With the loss of vasoconstriction at high intravascular pressures, arterial diameter will increase as will blood flow in accord with Poiseuille’s law. In SAH animals, cerebral arteries are more constricted at physiological blood pressures, thus have less capability to further constrict and increase arterial resistance as blood pressures increase. Therefore, cerebral arteries/arterioles in SAH animals may reach their maximum constriction/resistance at lower blood pressure compared to healthy animals. Once cerebral arteries/arterioles reach their maximum constriction/resistance, further increases in pressure would result in the passive enlargement of their diameter (i.e., “forced dilation”) leading to increased CBF and loss of cerebral autoregulation. Our finding that SAH shifts the upper limit of cerebral autoregulation toward lower blood pressures may help to explain the therapeutic efficacy of hypertensive intervention in SAH patients. In 1987, Awad et al. first described the “triple H therapy”—hemodilution, hypervolemia, and hypertension—to improve cerebral perfusion in SAH patients (Awad et al., 1987). Ensuing clinical trials demonstrated that “hemodilution” is unfavorable due to decreased oxygen-delivery capability (Chittiboina et al., 2011) and that “hypervolemia” did not improve patients’ outcome (Lennihan et al., 2000; Egge et al., 2001), and thus, triple H therapy is no longer used clinically with SAH patients. On the other hand, multiple studies have demonstrated that blood pressure elevation successfully improves cerebral perfusion in SAH patients and vasopressor agents are frequently prescribed once aneurysms are secured (Muench et al., 2007; Treggiari, 2011; Haegens et al., 2018). A better understanding of the cellular mechanisms contributing to the narrowing of the autoregulatory range after SAH may provide insights into therapeutic strategies to improve CBF without necessitating an increase in systemic blood pressure. Further, as chronic hypertension can impact CBF and is a risk factor for SAH, additional studies examining hypertension and SAH as comorbidities are warranted.

In conclusion, by developing and applying an approach enabling the quantitative analysis of the relationship between blood pressure and CBF, this study provides valuable insights into the detrimental impact of SAH on cerebral autoregulation. Of import, we demonstrate a downward shift in CBF across the range of autoregulatory blood pressures in SAH mice. Further, the upper blood pressure limit of cerebral autoregulation was reduced in SAH mice compared to sham-operated and un-operated animals. The resulting reduction in CBF combined with a narrower range of cerebral autoregulation likely contributes to DCI after SAH. Moreover, our newly established method of quantitatively analyzing cerebral autoregulation creates a foundation for future studies to examine physiological and pathological mechanisms of cerebral autoregulation in a host of disease states.

## Data Availability Statement

The original contributions presented in the study are included in the article/Supplementary Material, further inquiries can be directed to the corresponding author/s.

## Ethics Statement

The animal study was reviewed and approved by the Institutional Animal Care and Use Committee at the University of Vermont.

## Author Contributions

MK designed and directed the research, acquired and analyzed data of *in vivo* and *in vitro* experiments, and prepared the manuscript. HF supported *in vitro* procedures of absolute CBF measurement. MN contributed to the research management. GW designed the research and prepared the manuscript. All authors contributed to the article and approved the submitted version.

### Conflict of Interest

The authors declare that the research was conducted in the absence of any commercial or financial relationships that could be construed as a potential conflict of interest.
