# Supplementary material for: Impaired Cerebral Autoregulation After Subarachnoid Hemorrhage: A Quantitative Assessment Using a Mouse Model
Source: Front Physiol. 2021 Jun 8;12:688468. doi: 10.3389/fphys.2021.688468 (PMC8218876; doi:10.3389/fphys.2021.688468)
Supplement: Supplementary file 1 [file image_1.pdf]

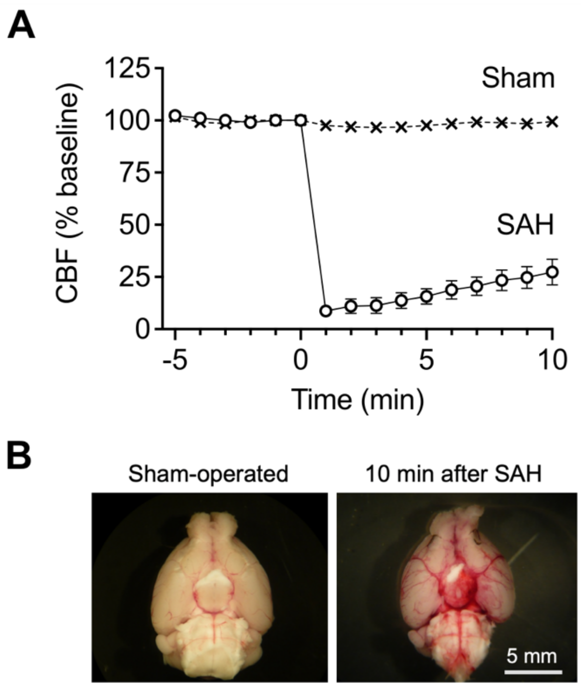

Supplemental Figure 1. Mouse endovascular perforation SAH model. **A**) Cerebral blood flow (CBF) monitoring during the induction of SAH or sham-operation. CBF was measured using a flexible glass fiber laser Doppler probe directly attached onto the intact skull. A 5-0 monofilament suture was inserted from the external carotid artery into the internal carotid artery and proceeded to the anterior cerebral artery-middle cerebral artery bifurcation. Further advancement caused artery puncture leading to SAH at a time of 0 min (n=13). No further advancement of the suture in sham-operated groups (n=10). **B**) Mouse brains from sham-operated and SAH model animals (10 min after surgical procedure).
